# Therapeutics

**Published:** 1825-01-01

**Authors:** 


### Therapœia.

Pharmaca nulla valent, nisi quæ sint commoda causis.

1. *Iodine.** The papers, of which the titles are given below, fell into our hands after the review of Dr. Gairdner's work was printed off, otherwise we should have included them in that article.

Dr. Kolley has been severely affected with goitre in his own person, and has taken iodine internally on a large scale, besides having employed the medicine in his own practice:—His observations are therefore entitled to consideration. He commences his memoir with some reflections on the indications and counter-indications in the employment of this powerful remedy.

*Contra-Indications.*

1. Iodine, he observes, in the form of tincture, harmonizes badly with the nervous system. In persons of much sensibility, even a small dose often gives rise to disagreeable accidents and violent spasms. They experience an inquietude in their limbs which prevents repose, succeeded by a sense of lassitude and weight—deafness—violent cephalalgia—tremors of the limbs—depression of spirits—moroseness—spasms—pallor of the face—lachrymation—great anxiety about the chest—and unconquerable aversion to the medicine. These symptoms are altogether different from those that result from a complete saturation with the iodine.

2. People who have a disposition to congestion of blood in the head or any other internal organ—whose constitutions are of the robust, muscular, and, what is termed, atrabilarious temperament, do not bear well the action of iodine—particularly if unaccompanied by laxatives. In such constitutions the tendency to local congestion augments—the head becomes embarrassed, and cephalalgia rises to such an extent as to threaten delirium.

3. In all cases where there is a deranged state of the digestive organs, great caution should be used in administering iodine, otherwise dyspepsia and other gastric affections will be increased or produced.

4. All febrile and inflammatory affections interdict the use of iodine, and the same may be said of diarrhœa—local affections of the urinary organs—phthisis pulmonalis—hepatic disorder. Neither should this medicine be incautiously given to those who eat and drink beyond the rules of temperance; else febrile excitement or hæmorrhage will be endangered.

---

* Reflexions et Observations sur l'Emploie de l'Iode en Médécine. Par le Docteur Kolley, Médécin à Breslau.—*Journal Comp. Fevrier*, 1824. *Observations* sur l'Emploi de l'Iode en Médécine. Par M Zink, Médécin à Lausanne.—*Ibidem*.

*Indications.*

5. Iodine may be safely employed where there is no morbid sensibility or irritability present in the system—where there is no tendency to congestion in any organ—where there is no gastric derangement, febrile affection, plethora from too full living, diarrhœa, &c.

Our author confines his observations to the internal use of iodine, not having experience of its external application. He considers this medicine as very powerful in goitre and scrofula—far superior to any other remedy which has yet been found out. But to employ it with success in goitre, it is necessary first, that the swelling be confined to the thyroid gland; 2dly, that the tumour has not degenerated into a scirrhous or carcinomatous nature—3d, that the bronchocele be not of too long standing—4th, that the local affection be not accompanied with derangement of the general health—fifthly, and lastly, that the goitre be not inflamed or very painful. The above objections apart, it is very rare that we fail of success in removing bronchocele by iodine.

This medicine possesses an extraordinary power over scrofula, provided there be no strong tendency to phthisis, which iodine would be apt to aggravate. When administered to children, the regimen should be very severe, and they should be kept as much as possible in a regulated temperature, and there have some exercise. Where the constitution of the child is irritable, or there is any fear of hydrocephalic affection, this medicine should be exhibited with the greatest caution, or, rather, it should be abstained from altogether. Our author has found it very useful to combine this medicine with a gentle mercurial, particularly where the disease has continued for some years, as in scrofulous ophthalmia. In such cases it is proper to intermit the iodine occasionally, continuing the mercurial in the intervals.

Perceiving, in common with other practitioners, that iodine has the property of modifying the assimilation, or rather of exciting powerfully the lymphatic and absorbent systems, our author was led to its employment in certain chronic diseases, and derived great benefit from it in herpetic eruptions of the skin.

Dr Kolley, being himself affected with goitre for ten years, and having tried all the means recommended by authors without success, was induced to have recourse to iodine, as soon as Dr. Coindet's Essay appeared. At this period, his respiration was very much impeded—he could not walk fast nor far—it was even difficult for him to go up stairs, or speak for any length of time. In addition to these symptoms, he was affected with much congestion of blood about the head. He took 10 drops of the tincture of iodine, as recommended by M. Coindet, three times a day, for a fortnight, without experiencing any inconvenience. During the succeeding fortnight he experienced an augmentation of the gland, with a corresponding aggravation of all the symptoms abovementioned. He also felt a disagreeable taste in his mouth, diminution of appetite, but without any alteration in the secretions or excretions. After this the gland began to diminish, and in twelve weeks had almost entirely disappeared. During the whole of that period, our author continued the internal use of the iodine uninterruptedly. He experienced no other inconvenience from the remedy than a diarrhœa for a few days, when he had raised the dose of the tincture to 40 drops. The venereal appetite ceased during the use of the medicine; but our author experienced no emaciation, nor any of the bad consequences enumerated by Coindet and others. This, however, he ascribes to idiosyncrasy, and does not mean to say that such immunity is always to be expected, when the medicine is carried to the extent in which it was used in his own case. For instance, he prescribed the tincture to a female, 45 years of age, of irritable constitution, and long affected with hysteria and spasms. She could not take doses of three or four drops without experiencing great anxiety, tremors, cold sweats, nausea, and agitation.

The combination of iodine with mercury and tonics has been found very efficacious by our author in scrofulous affections, especially of children.

Of the eleven cases which are related by our author, in elucidation of the preceding observations, our limits will permit us to take but a short notice.

*Case* 1. A peasant girl, 18 years of age, had had an increasing bronchocele for the last five years. It was now of great size, with a sulcus in the middle, and occasioned considerable embarrassment of the respiration. It was hard, tense, and covered with dilated veins. The digestive and other functions were not deranged—nor was there any congestion about the head. Six drops of the tincture of iodine were ordered thrice a day, enjoining a very abstemious regimen. At the end of fifteen days the goitre was rather increased in size, and the breathing more difficult. The dose of the medicine was augmented to eight drops. During the next fortnight, there was a notable alteration in the tumour. It had diminished in size—was less tense—and its vessels less varicose. These changes were still more considerable by the end of six weeks from the commencement of the plan of treatment. It was persevered in for sixteen weeks, with great diminution of the goitre. At this time, inappetency, head-ache, nausea, and vomiting came on. The iodine was discontinued, and the consequences, for some days, carefully watched. The patient returned into the country, and, three months afterwards, presented herself to our author, pale and emaciated, with great enlargement of the abdomen. This last, however, was ascertained to be pregnancy. The goitre had almost entirely disappeared. The young lass was safely delivered of a healthy child at the proper period.

*Case* 2. A man, 36 years of age and otherwise healthy, had been goitrous from the age of sixteen. When examined by our author, the tumour was large, uniform, and solid, with some elasticity. It caused great difficulty of breathing, wheezing at each inspiration, cough, and alteration of the voice, with congestion about the head. Six drops of the tincture of iodine were prescribed three times a day, and a laxative twice a week. This plan agreed very well. The goitre diminished fast, as did the difficulty of breathing. At the end of eight weeks the tumour was so much reduced that the iodine was discontinued, the patient beginning to complain of gastric affection, which disappeared in a few days afterwards.

*Case* 3. This was a case of goitre in a young girl. When she had taken the iodine for three weeks, she suffered such anxiety, lassitude, burning in the throat, &c. after each dose of the medicine, that she refused to continue it any longer. The bronchocele, in the mean time, was beginning to diminish sensibly.

The remainder of the cases contained in this memoir, are those of scrofulous affections cured by iodine, in conjunction with mercury. These we must pass over, as not presenting unequivocal proof of the particular efficacy of iodine, though we have no doubt that it is possessed of considerable power over scrofulous as well as other morbid swellings.

We now come to Dr. Zink's cases, where the hydriodate ointment was used.

*Case* 1. A young female child, five years of age, was brought to our author, with a white swelling of the left ancle, which occasioned great pain when she attempted the least motion. The tumour did not surround the whole of the joint, being in two divisions, one at each malleolus. The skin had not changed colour, though it was tense, shining, and elastic. Wishing to ascertain precisely the effects of the iodine ointment, our author applied it night and morning to one side of the tumour. In the course of a month that part of the swelling had almost entirely disappeared, and was devoid of pain. The frictions were now only continued once a day, and in three months the swelling on the opposite side disappeared also.

Before proceeding to the second case, our author makes some observations on the internal use of iodine. He had an opportunity of seeing two fatal cases from the effects of this medicine, and of examining the bodies after death. The particulars are reserved for a future paper. In the mean time, he recommends the external use of the remedy, in preference, and we join him in this recommendation.

*Case* 2. An unmarried lady, 27 years of age, in good health otherwise, had, for a considerable time, a tumour in the right arm-pit, the size of an egg; apparently an enlarged lymphatic gland. It was indolent, hard, unequal, and moveable. Although not tender to the touch, it gave much inconvenience from its size, and by its pressure on the axillary nerves. Another indolent tumour was found situated two inches above the inner condyle of the humerus. The iodine ointment was commenced on the 25th January, 1822, and continued till the beginning of April. The tumours were dissipated, but not without some suppuration taking place.

*Case* 3. A young lady had a hard, indolent tumour in one of her breasts, remaining after a contusion of the part, and now becoming painful. Leeches and cataplasms were first applied, and then the iodine ointment. The ointment did not appear to take any effect for some time on the mammary swelling, but, in a few days, it completely dissipated a large goitre which she had on one side of her throat. Leeches, poultices, and gentle purgatives, now removed the tumour in the breast.

We have lately had a demonstrative proof of the efficacy of iodine ointment on an indolent and very hard tumour in the breast of a young lady, which had resisted every other means, and speedily gave way to two or three boxes of the ointment.

---

2. *Dangers of Iodine.* A considerable number of practitioners in this metropolis have now exhibited iodine internally, and we have not been able to learn that any of them met with sinister accidents from its exhibition. Several of them have, in consequence, begun to doubt the fidelity of the statements which have reached us from the Continent, and have expressed themselves to be sceptical as to the reality of the dangers which we have more than once pointed out in this Journal, founded on events which have occurred on this side of the Channel. It requires but a short retrospective glance at the history of active medicinal substances, to be convinced that, from some cause or other, their good effects are magnified, and their dangerous qualities concealed, at the beginning—and that a considerable period of trial is often necessary, to bring the merits and demerits of a remedy to the proper level of truth. Under this conviction, we shall go on with details of the success and danger of iodine, as they may reach us through public prints or private channels.

Dr. Miguel, Editor of the GAZETTE DE SANTE, relates the following case, which happened in his own practice.

Alexandrine C——, 17 years of age, had a large goitre, and was sent to the author for advice, by Dr. David, of Meulan. At first, the iodine ointment was tried, but without much sensible diminution of the tumour. Dr. Miguel then gave the patient some tincture of iodine, desiring her to take six drops three or four times a day, and not to exceed this quantity, without advice. She disregarded his injunctions, however, and not leaving her address, Dr. M. lost sight of her for nearly three months. He then received the following account from Dr. David, her original physician.

"Alexandrine, anxious to get rid of her goitre, exceeded your directions, and took the tincture in too large doses, without my consent. The consequence was, that the menses ceased, the pain in her stomach increased, and she was, at length, obliged to take to her bed, where she was found by the medical man of the house where she lodged, in a most deplorable condition. It was not till after some weeks that she was able to be moved, and came back under my care. Her complexion was now of a deadly pallid hue—her eyes without any other expression than that of severe suffering—voice nearly extinct—pulse scarcely to be felt, though incredibly quick—skin cold, but not dry—extreme prostration of strength—cephalalgia—entire loss of sleep—occasional delirium—constant spitting, amounting to a kind of salivation—small, dry cough—voracious craving for food and drink, which were rejected from the stomach as soon as swallowed—pain and extreme sensibility over the epigastric region—constipation—high-coloured urine—tension of the abdomen, &c."

These symptoms, clearly indicating irritation, if not chronic inflammation, of the mucous membrane of the alimentary canal, demanded rigorous and abstemious regimen—leeches to the epigastrium—the tepid bath—emollient drinks, &c. By these means, the gastro-intestinal irritation was gradually, though slowly, removed, and the patient restored to tolerable health. The volume of the goitre was reduced about one third, and rendered more soft.—*Gazette de Santé*, *Mars*, 1824.

---

3. *Dysentery.* In hot climates, dysentery often occurs in such an extremely acute form as to be justly considered a violent congestion or inflammation of the mucous membrane of the intestines, and, in such instances, requiring the most decided depletion. Such was the dysentery described by Dr. O'Halloran in a late number of the Medical Repository, as occurring at Gibraltar among the young men of the 64th regiment. The patients had a constant inclination to stool, but only voided blood and mucus—the former frequently in great quantity. In three fourths of the cases, there was high febrile action—and many of them had great pain and difficulty in making water. This was a dangerous symptom. The mode of treatment adopted was simple, and appears to have been eminently successful. It consisted principally in early, copious, and repeated venesections, with the daily use of the sulphate of magnesia. From thirty-two to sixty-four ounces of blood were usually taken at the first bleeding. If faintness did not then ensue, the patient was raised, and the change of position generally induced syncope. The average quantity of blood drawn from dysenteric patients, in the course of the treatment, ranged from five to eight pounds—a quantity ordinarily taken away before the termination of the second day. Scybalæ were rare, either in spontaneous evacuations ,or those produced by medicine. Sometimes, a typhoid fever succeeded the dysenteric symptoms. "For the removal of it, calomel and James's powder, five grains of the former to three of the latter, were given thrice a day, and the patients became convalescent in a short time, soon regaining strength."

Dr. O'Halloran must be aware, that the same plan of treatment was pursued in the army during the Peninsular war; and that, for 20 years past, bleeding has been employed in acute dysentery by our tropical practitioners.

---

4. *Striking* CURES. All the world has now heard of the striking cures performed by Dr. Balfour of *percussive* celebrity. Gout and rheumatism have been so *pummelled* by the Doctor, that they dare not shew their faces in the intellectual city. In fact, he seems determined to *strike* them off the nosological list of his immortal countryman—Cullen. These formidable enemies being placed *hors de combât*, the Doctor has attacked a still more rebel class—the *neuroses*, which appear, also, to fly before his powerful hands. The 34th case, lately published in the Medical and Physical Journal, affords a remarkable example of Dr. Balfour's success. The patient, an elderly lady, had the vapours in an extraordinary degree, being sometimes so oppressed in her spirits and feelings, "*that she could neither lie, sit, stand, speak, or listen to others!*" This being the case, we confess we are somewhat at a loss to imagine in what position the Doctor fixed his patient for the operations of percussion and compression—unless it was in that of flying or swimming. He did manage, however, to apply "percussion to the spine, shoulders, and superior extremities." "The effects were an improvement of feeling and a buoyancy of spirits, far exceeding her expectations" "I explained to her," says the Doctor, "the principles of my practice, *which she readily comprehended*, and considered the information as a most important acquisition." We hope Dr. Balfour's readers will comprehend his principles with the same facility as the old lady, who could "neither speak nor listen to others," and that the information contained in this endless series of cases, will be considered by them also, as "a most important acquisition."

---

5. *Poisoning by Opium.* Dr. John Crampton has published some cases of this kind in the 4th Volume of the Dublin Transactions. The two first cases were fatal, and need not be detailed. In the third, our author was more fortunate, and the success is ascribed to the plan first promulgated by Mr. Wray and Dr. Copland, viz. the affusion of cold water.

*Case.* In February, 1823, Dr. C. was called to a lady who had swallowed about two ounces of laudanum, at 8 o'clock that morning, an hour and a half before his arrival. An apothecary had given her sulphate of zinc and chamomile tea, endeavouring to excite vomiting by a feather. Our author exhibited about two drachms of tartar emetic in divided doses, but without any effect. She evinced much propensity to sleep; and this was prevented by placing a cold wet cloth on the naked scalp, and by sprinkling cold water on the face, which seemed to rouse her sufficiently. She was next placed in an open carriage, her head freely exposed to the cold air, while aspersions of cold water were practised from time to time by her female attendants. This process our author superintended personally, and had the satisfaction to find that copious vomiting soon commenced. "The stomach, in fact, appeared to regain its natural susceptibility to emetics, under the joint influence of the cool air, the cold aspersion, and the motion of the carriage." In the course of an hour the patient was so far relieved that Dr. C. took his leave.

"In the event then of poisoning with Opium, if the above views are correct, our practice should be, to use emetics without loss of time, shave our patient's heads, expose them freely to cold air, and make use of aspersions or affusions of cold water, availing ourselves of the advantage of exercise in some open vehicle. Mr. Jukes' improved tube and syringe, for emptying the stomach mechanically, might also be used with advantage, provided it was resorted to at an early period after the opium had been swallowed, and before the full narcotic influence of the poison had been exerted on the vital powers of the constitution." 302.

---

6. *Pneumonia.** Dr. O'Halloran, the intrepid investigator of the Spanish and West-India fever, has given us an hospital report on Pneumonia, from the 64th regiment, stationed at Gibraltar. His treatment of this disease was bold and decisive—and, as far as Europeans were concerned, remarkably successful. In all cases of this disease he very properly placed his chief reliance on the lancet for the removal of the local inflammation, aiding this powerful measure by purgatives, nauseating doses of antimonials, and the *warm bath*. This last, however, was only used on the admission of patients, and very rarely afterwards. If the symptoms were urgent and the patient young and robust, from 32 to 64 ounces of blood were abstracted at once, and if syncope did not take place in the recumbent posture, it was changed for the perpendicular, when fainting usually ensued. It was often necessary to repeat the operation a second, and sometimes a third time in the same day. After the first bleeding, ten, fifteen, or twenty grains of calomel, combined with ten grains of extract of colocynth, were exhibited, and even this strong purgative often required salts to expedite its action. On the second day, if the distress of the lungs had not abated (and this was often the case) the bleeding was repeated, and tartarized antimony, in nauseating doses, was given. Our author had rarely occasion to bleed on the third. The antimony was continued, and the patient usually was up by the fourth or fifth.

The above practice is not new in the Army and Navy, nor is it unknown to private practitioners. But it is rarely put in force by the latter. Perhaps it would not be either prudent or proper to carry measures with so high a hand in private life. Many objections would be made to it—and many impediments thrown in its way. On the other hand, there are many auxiliaries to the above powerful measures that render it, in the majority of cases, unnecessary to push them to the extent described by O'Halloran. Add to this that, in private practice, we have more deteriorated constitutions and less simple forms of disease to manage than among the class of patients under O'Halloran's care.† Under all these circumstances, the young practitioner will do well to

---

* Med. Repos. No. 8.

† "The regiment is principally composed of young men, from seventeen to thirty years of age."—*O'Halloran.*

view the above plan of treatment as one which may *occasionally* be adopted among the young and vigorous of his patients, but not put commonly into use among the general run of constitutions.

---

7. *Diabetes.** We suspect, with Dr. Sharkey, that we have been a little out of our reckoning, of late years, respecting the diet of diabetic patients. Animal food, we much fear, has no very great effect in checking this formidable disease, even when rigidly adhered to. But the fact is, that few or no patients will confine themselves to a strict diet of animal matters. We were lately in attendance on two diabetic patients where the animal food system was pursued for some time, without the least advantage. Both patients got tired of this system, and indulged freely in vegetables without any increase, but rather a decrease of the diabetic urine. The compound powder of ipecacuan and superacetate of lead gave most relief. They ultimately relaxed the skin and checked the inordinate secretion of saccharine water.

Dr. Sharkey met with two cases of diabetes mellitus in the years 1812-13, where the treatment of Rollo was first used without advantage, and where a different treatment succeeded. The treatment may be described in a few words, as it consisted principally in the administration of phosphate of soda—a remedy which the elder Dr. Latham, indeed, had used in similar cases before, but only as a subordinate auxiliary. Dr. S. was led by accident to the employment of this medicine, from observing a remarkable diminution of urine after taking a dose of the salt as a common purgative.

In one of these cases we perceive Dr. S. used previously the superacetate o flead and opium, employing the phosphate of soda to counteract constipation at first. Under these circumstances, a doubt may arise whether the cure was attributable solely to the phosphate. Dr. S. also gave with it the decoction of cinchona. The dose was generally a drachm of the phosphate thrice a day. In the second case, however, he informs us that the phosphate of soda *alone* was employed, and "the disease terminated favourably in a short time."

In a complaint which so frequently baffles every medicine the foregoing hints may be acceptable.

---

8. *Sulphate of Quinine.*† The success of sulphate of quinine in intermittent fevers naturally leads to a confident expectation that its powers will be extended to many other diseases—and that its *febrifuge* qualities may not be confined to a single class of fevers. The minute-

---

* P. Sharkey, A.M. M.D. Physician to the Cork General Dispensary. Dublin Transactions, vol. iv. p. 379, *et seq.*

† On the Effects of Sulphate of Quina in Typhus. By John O'Brien, M.D. &c. Fellow of the King and Queen's College of Physicians in Ireland. *Dublin Transactions, vol. iv. p.* 367—378.

ness of its dose, the facility of its exhibition, the convenience of its form, and its levity on the stomach, seem to promise an agent possessing all the virtues, and few or none of the inconveniences of the Peruvian bark.

In conformity with these views, Dr. O'Brien felt it his duty to give the medicine a trial in a few cases of typhus. The results have impressed him with a very favourable opinion of its efficacy, under certain restrictions, in this class of fevers.

In the months of October and November, 1823, a great influx of fever cases took place into the Fever Hospital, Cork Street, of rather a malignant type.

"This fever was in general characterized by large petechiæ, of a dark bluish colour, in the form of maculæ or blotches, variegating the whole surface of the body in a very singular manner. Four individuals of a family, named M'Gowan, in different wards of the hospital, exhibited a skin covered from head to foot precisely in the same manner with the petechiæ just described. The fever seldom terminated before the fourteenth or fifteenth days, but was frequently protracted to the twentieth and twenty-first. The organs principally affected were the brain and nervous system, evinced by delirium in all its degrees, muscular tremors, subsultus tendinum, &c. pulmonary congestion or inflammation were also frequent. In the most malignant and fatal cases, both the head and chest jointly exhibited the usual marks of congestion, or inflammatory action; a combination of symptoms to which the petechiæ above-mentioned were generally superadded. From the class of cases just described, six were selected indiscriminately, for trying the effects of the sulphate of quina." 369.

In two cases, the effect was as decisive, and the recovery as rapid, as in intermittent fever. In three cases, the result was less rapid, but not less effectual.—The patients improved gradually, and ultimately recovered without relapse. One patient out of the six died. On an impartial review of this last case, our author cannot consider it as a decisive instance of failure in respect to the medicine.

"It presented a specimen of that intense and complicated form of typhus, the most formidable we encounter in our hospital, in which there exists a constant tendency to inflammation or congestion simultaneously in several organs essential to life, but principally in the brain and lungs, whilst the failure of the strength and vital powers forbids those measures of depletion necessary to restore the balance of the circulation." 371.

In most instances of this kind, there exists a predisposition in the organs, probably some structural lesion, which leads to inflammation or congestion, during the orgasm of fever. This appears to have been the case of the patient who died.

"On his first admission, which took place about the fifth day of this fever, as well as I could learn, the prominent symptoms which presented themselves, were those of mild pneumonic typhus, (pneumonia typhodes of Dr. Cullen,) viz. a quick and rather feeble pulse, dull pain and oppression of chest, accompanied by a wheezing respiration, foul tongue, and clouded skin; but no petechiæ appeared till a more advanced stage of the disease." 371.

The local affections were treated in a judicious manner, and, on the 13th day of fever, the sulphate of quina was commenced, with the view of giving tone to the vascular system, and counteracting its approaching collapse. It was continued four days, in doses of four grains per diem. The first effects observed were highly favourable. The pulse became slower and firmer—the countenance exhibited more energy—the petechiæ became more full and florid—and the tongue became clean and moist. This favourable state of things was not permanent. On the 17th day, the respiration became laborious and sonorous. The quina was discontinued, and cordials and stimulants employed. Death took place on the 21st day. This case certainly affords no evidence unfavourable to the sulphate. Indeed, it is doubtful, as Dr. O'Brien observes, whether the event might not have been more fortunate, had the quinine been persevered in. We see no possible reason for substituting *cordials and stimulants* for the quinine. The *latter* medicine was the best cordial and stimulant they could have given—and if *it* was improper, so must have been those medicines subsequently exhibited.

The two patients who had rapid recoveries, were both affected with "fever of a severe and dangerous character"—both having petechiæ and blackness of the tongue. There were no appearances of a remittent type in either case. Both patients were of a thin and delicate frame, and a weak and flaccid muscular fibre—circumstances unquestionably favourable to the quinine.

Every one knows that the bark used formerly to be given in typhus, as well as in every other kind of fever. We are informed by Morton, that one of the arguments urged by the opposers of this medicine, was, that its introduction must destroy altogether the profession of physic, as every man would then enjoy health without the aid of the Doctor! An argument founded on such ignorance and self-interestedness, would excite no small degree of surprize in these days. It is, therefore, to be hoped, that we are both wiser, and more liberal than were our forefathers one or two centuries ago.

The failure, and consequent disuse of bark, doubtless arose, as Dr. O'Brien observes, from the indiscriminate and injudicious manner in which it was administered, as well as from the inconvenience of its bulk. Indeed, this last disadvantage renders bark inadmissible in the great majority of continued fevers, where nausea and irritability of stomach so generally prevail. From this draw-back, the quinine is altogether free, as it very rarely disagrees with the stomach.

Dr. O'Brien infers, from the foregoing cases, that three or four grains *per diem* will be generally sufficient to insure the success of the remedy—and perhaps even smaller doses.

"With respect to the stage of fever most appropriate for commencing the Quina, the period of direct debility or collapse appears to me the most suitable. In the stage of excitement it must be remembered, that one or more organs are found to labour under inflammation or congestion; and the stomach, participating in the general disturbance of the nervous and vascular systems, is weak and irritable. To such a state of things the quina is inapplicable. If administered under such circumstances, its effects will be, to augment the heat of the skin, parch the mouth and embrown the tongue, promote costiveness, and increase the errors of the circulation. When the period of excitement has passed away, the temperature of skin fallen, and the circulation in some degree restored to an equal balance, we may safely commence the Quina. The moment, in fine, when the cordial treatment becomes necessary, seems to be the most proper for calling to our aid the tonic powers of the Quina." In fever of a remittent type, I apprehend less caution may be necessary. 377.

We are convinced, as we stated in a former number, that the sulphate of quinine will soon become applicable to a great many complaints where no form of bark is now considered admissible.

---

9. *Hospital Reports, from La Charité under Professor Laennec, for the first quarter of* 1824. (Revue Medicale, Mai, 1824.)

This report is made out by Meriadec Laennec, "Aide de Clinique," and the son, we suppose, of the celebrated Professor. The entries were 222 cases, of which one hundred and twelve were acute diseases—eighty-six were chronic maladies—and twenty-two valetudinarians. The acute diseases were fevers, the various phlegmasiæ—exanthemata—rheumatisms, &c. More than half the cases of fever were mild, and occasioned little alarm. Four died out of 36—or one in nine. This, we think, is a pretty high ratio of mortality, considering that there was no epidemic, and so large a proportion of mild cases. Three of the four who died were young men, and the fourth a boy of 15 years of age. All the fever cases were treated "*par la méthode expectante.*" A few leeches were applied in the beginning, when there existed any signs of gastro-intestinal irritation or cerebral congestions, with diluents, and rigid regimen during the acute stage of the disease. We have given, in the first article of this Number, M. Laennec's ideas respecting the connexion of fever and inflammation—and need only say here that he considers the disease as no way dependent essentially on local inflammation; for, when the latter exists, the fever is symptomatic—in short, that inflammation is a mere contingency that may or may not take place in the course of idiopathic fever. Of the contingent inflammations he has found those of the thorax the most frequent. The sanguineous *congestions* which we observe in dead bodies, take place, he thinks, for the most part, during the agonies, or even *after* death. He thinks that medicine has little control over the march or duration of fever—although it has often the power of combating the contingent phlogoses, and thus saving the life of the patient.

Of intermittents, two cases only occurred, and were cured by the sulphate of quinine, in doses of 18 grains in 24 hours. Of five apoplectic attacks, three died, and two remain hemiplegiac.

There were 28 cases of pleurisy, peripneumony, and pleuro-pneumony. Peripneumony was recognized, in every instance, from the very beginning, by means of the stethoscope. Only one died, although, in several of the patients, the lungs of both sides were affected. In two cases there was a total absence of the respiratory murmur throughout one whole lung, and half the other—so that three-fourths of the pulmonary apparatus had become hepatized.* Five or six shewed unequivocal signs of pulmonary abscess. Under these circumstances the loss of only one in the 28 cases speaks for the merits of the method of treatment. This method was by large doses of the tartrite of antimony, as we have already made known in a former Number of this Journal. "In one patient, who was very weakly, the pectoriloquism and cavernous rattle (râle caverneux) indicated the existence of two abscesses in the right lung. The emetic tartar was given in large doses (nine grains in the day) and was well borne. The inflammatory orgasm was arrested. The dose was carried, in another case, to 12 grains, and afterwards to fifteen in the 24 hours. Both patients became rapidly convalescent."

There were five cases of acute articular rheumatism, which all gave way to antimonials administered in large doses.

Among the cases of phthisis which shewed unequivocal evidence (by the stethoscope) of pectoriloquism, or tubercular excavations, this symptom became stationary in three cases—the patients gathered flesh and strength, coughing and spitting very little. M. Laennec regarded these as instances of cured phthisis—but thinks that, at some future day, fresh tubercles will suppurate and destroy their lives. Phthisis is so easily ascertained by the stethoscope, that the pupils themselves, in a very little time, had no difficulty in recognizing it. Even crude tubercles, and in no great number, were often detected by mediate auscultation.

Eight cases of disease of the heart presented themselves this quarter, of whom three died—all with hypertrophy. One very old man was *in extremis*, when he entered the hospital, and the disease was evident. But the extent of the hypertrophy was not suspected even by Laennec with the stethoscope in his hand. The heart was nearly as large as the man's own head—while the sound and impulsion, during life, were not at all in proportion to this astonishing magnitude. In the cases of those who have yet survived, the abstemious and depletory plan of Valsalva had some effect in mitigating the symptoms. One young man, 18 years of age, had been affected with slight acute rheumatism, which gave way in a few days to tartarized antimony; (six grains in the 24 hours.) On the 5th day of convalescence, he shewed some symptoms of very slight pleuro-pneumony, and M. Laennec accidentally examined the chest with the stethoscope, when, to his surprise, he found the action of

* Does M. Laennec mean to say that *these* recovered! They might not die within the date of the report, but we believe it to be absolutely impossible for a hepatized lung to recover its pristine organization, or for a patient with such disease to completely recover health.—*Rev.*

the heart extremely violent, and he had no hesitation in pronouncing the melancholy fiat—hypertrophy of the heart. Yet, in this case, there had been no disturbance of the circulation or respiration, or any symptom that could authorize the suspicion of disease of the heart going forward. The pulmonic inflammation yielded to two bleedings, the application of leeches, and the use of antimonial powder, 36 grains *per diem.* But the general signs of hypertrophy of the heart developed themselves daily more and more. Latterly the contractions of the heart are become tumultuous, accompanied by an obtuse pain in the cardiac region, where the sound has become dull and almost inaudible—signs which seem to announce the existence of pericarditis. Finally, this patient presents a hissing noise in all the arteries of the body—a phenomenon which M. Laennec considers as no proof whatever of disease in any of the arteries. At the same time, when it obtains in many arteries at once, it is a symptom of danger.

Seven cases of peritonitis, chronic or subacute, occurred, of which four were completely cured by mercurial frictions. "The moment the mouth became affected the peritoneal inflammation ceased." "Dans les quatre cas indiqués, ce traitement a été tres efficace, *et dès les premiers signes de salivation, les symptomes de peritonite ont constamment desparée.*" This will set the hair of the whole French faculty on end. Mercury to cure a chronic inflammation! They will not believe it, even from the mouth of Laennec. He took the idea from Mynheer Vandinzande of Antwerp, but it is evident enough that the practice has been long familiar to English physicians.

Seven cases of sciatica occurred, and were all cured by the plan long ago recommended by Cotugno—viz. blisters kept open for several weeks, not near the origin, or in the course of the sciatic nerve, but as near as possible to the extremities of the nerve. Thus, a blister below the head of the fibula (dessous de la tête du peroné) or on the instep, and kept open for some time, will often cure a sciatica that has resisted repeated blisters higher up. The suppuration of the blister should be maintained for six weeks at least. For twenty years past M. Laennec has never known this treatment fail. There was a curious case where a continued pain emerged from the last dorsal and first lumbar vertebræ, terminating in the linea alba. Considering the affection as neuralgia of the inferior dorsal and upper lumbar nerves, M. Laennec applied a long blister on each side of the linea alba, and kept it open for five weeks, when the patient was completely cured. This hint is worth attending to in other neuralgiæ.

In two cases of incomplete amaurosis, the ammoniacal pomatum of Gondret was employed with success, by being applied to various points of the vertex.

We shall be happy to see a continuation of these Hospital Reports from so excellent a pathologist as M. Laennec.

